# The Acceptability and Feasibility of Self-Collected HPV Testing for Cervical Cancer Screening Among Black and Latinx Women in Chicago: Perspectives from the Community

**DOI:** 10.1089/whr.2024.0102

**Published:** 2024-09-30

**Authors:** Emilie Glass-Riveros, Kelley Baumann, Katherine Craemer, Stacie Geller, Monica Nava Frenier, Jada McDonald, Hunter K. Holt

**Affiliations:** ^1^ Center for Research on Women and Gender, University of Illinois Chicago, Illinois, USA.; ^2^ Department of Epidemiology and Biostatistics, University of Illinois Chicago, Illinois, USA.; ^3^ Department of Obstetrics & Gynecology, University of Illinois Chicago, Illinois, USA.; ^4^ Department of Psychiatry, University of Nebraska Medical Center, Omaha, Nebraska, USA.; ^5^ Department of Internal Medicine, University of Illinois Chicago, Illinois, USA.; ^6^ Department of Family and Community Medicine, University of Illinois Chicago, Illinois, USA.

**Keywords:** HPV test, HPV testing, self-collection, self-swab, cervical cancer screening, cervical cancer

## Abstract

**Introduction:**

Cervical cancer disproportionally affects Black and Latinx women in Chicago. Black and Latinx women have a higher incidence of cervical cancer diagnosis and lower rates of cervical cancer screening than non-Latinx White women. Self-collected high-risk human papillomavirus (HPV) testing has been proposed as a method to address these barriers to screening and prevent cervical cancer.

**Objective:**

This study aimed to understand the feasibility and acceptability of self-collected HPV testing as a novel approach to address barriers to cervical cancer screening for Black and Latinx women in Chicago.

**Methods:**

Semistructured interviews with 17 Black and Latinx community members of the greater Chicago area were conducted. Thematic analysis using inductive and deductive coding was completed.

**Results:**

Findings from qualitative interviews indicate strong support for self-collected HPV testing among community members. They expressed a preference for self-collected HPV testing due to the comfort, control, and reduced anxiety it offers. Financial constraints, prioritization of other life demands, and past trauma were identified as substantial barriers to traditional cervical screening.

**Conclusion:**

Self-collected HPV testing could address barriers to cervical cancer screening by providing a less-invasive, patient-centered alternative to traditional methods. Self-collected HPV testing should be made accessible, be integrated into existing cervical cancer screening programs, and be covered by health insurance.

## Introduction

Cervical cancer screening is a highly effective at preventing cervical cancer and is recommended by the U.S. Preventive Services Task Force.^1^ Despite the wide availability of cervical cancer screening in the United States, Black and Latinx women are disproportionately burdened by cervical cancer incidence and mortality compared with non-Latinx (NL) White women.^2–4^ In Chicago, the cervical cancer incidence rate (cases per 100,000 women) is 12.12 for Black women and 9.08 for Latinx women, compared with 7.30 for NL White women.^5^ These disparities are especially pronounced in community areas that have experienced historic segregation^6^ and are predominately populated by Black and Latinx people on Chicago’s south and west sides, where the cervical cancer diagnosis rates are more than triple the 2021 national average (7.6 cases per 100,000 women).^5,7^

These disparities may be attributable, in part, to differences in cervical cancer screening prevalence. As of 2021, screening rates for Black (61.5%) and Latinx women (52.8%) are lower than the prevalence for NL White women (70.8%) in Chicago.^5^ Community areas in Chicago with a high burden of cervical cancer diagnosis have relatively low rates of screening.^5^ Rates of cervical cancer screening are also falling nationally; a recent study found a decrease in up-to-date screening from 86% in 2005 to 77% in 2019.^8^ These falling rates may be due to several factors including barriers to in-person care,^9^ gendered racism within the health care system that leads to fear and mistrust among Black and Latinx women,^10,11^ and the residual effects of the COVID-19 pandemic.^12,13^

Currently, the U.S. Preventive Services Task Force and American Cancer Society recommend screening for women age 30–65 every five years with primary high-risk human papillomavirus (HPV) testing as a screening strategy.^1,14^ Unlike cytology, HPV specimens may be collected from the vagina rather than the cervix, reducing the invasiveness and discomfort of screening and making self-collection possible.^15^ A meta-analysis found that self-collected specimens for HPV testing are as effective as clinician-collected samples for cervical cancer screening, provided that the PCR-based HPV tests are used.^16^ HPV self-collection has recently been approved by the U.S. Food and Drug Administration (FDA) for cervical cancer screening.^17^ In addition, efforts by the National Institutes of Health’s National Cancer Institute will likely lead to an integration of this method into guidelines and recommendations.^18,19^ It is critical to understand how to implement and utilize self-collected HPV testing in a variety of settings.

Self-collected HPV testing has improved uptake of cervical cancer screening outside of the United States, including hard-to-reach populations.^20–23^ Several methods of inviting women to participate in self-collected HPV testing programs have been studied, including sending test kits directly to women *via* mail-to-all or opt-in designs,^24–26^ community-based and social media outreach education campaigns,^27,28^ and door-to-door interventions with community health workers who deliver self-sampling kits to women in their homes or workplaces.^29–31^ However, uptake rates have been lower in high-income countries when the local context is not considered in the implementation of self-collected testing programs.^32^

Little is known about whether self-collected HPV testing could improve up-to-date cervical cancer screening rates among under- or never-screened Black and Latinx women in Chicago, or the best way to implement such practice. Because of the large differences in screening prevalence across Chicago’s neighborhoods and between racial and ethnic groups, culturally sensitive and tailored approaches are required to address barriers to screening among different populations. This study aimed to evaluate the acceptability and feasibility of self-collected HPV testing for cervical cancer screening among Black and Latinx women in Chicago using qualitative methods.

## Materials and Methods

### Interview guide development

Semistructured qualitative interviews were conducted from January to March 2023 with self-identified Black and Latinx individuals across the greater Chicago area. The interview guide (Supplementary Data S1) was designed to elucidate women’s perspectives on current methods of cervical cancer screening and understand if self-collection is acceptable and capable of potentially addressing previously identified barriers. During the interview we specifically discussed (1) previous experiences with cervical cancer screening and barriers to care, (2) what self-collected HPV testing is and how it is performed, (3) how self-collected testing could address previously explored barriers, (4) the best ways to implement testing in the future, and (5) how to communicate self-collection test results back to patients. The interview guide was developed using the research team’s clinical experience and the FRAME-IT approach: Each section of the interview guide followed the main objectives of the approach (*e.g.*, addressing feasibility, acceptability, and implementation from the perspective of the patient).^33^ Demographic questions (age, gender identity, and race/ethnicity) were also collected.

### Recruitment and eligibility

Eligibility criteria for this study included: individuals with a cervix who were 21 years of age or older, spoke English, currently living in the Chicago metropolitan area or one of its suburbs, and self-identified as Black/African American and/or Latinx. Individuals with a previous history of total hysterectomy were excluded from this study. The research team collaborated with clinical and nonclinical community-based organizations with established connections to these populations. The study particularly aimed to recruit participants residing in communities with low cervical cancer screening prevalence. Recruitment materials, flyers, and information sheets were disseminated in various community settings, including clinic waiting areas, community meeting spaces, and through email or text messages. To facilitate easy participation, interested prospects were able to scan a QR code to access study details and provide their contact information.

Participants underwent a screening survey, utilizing Qualtrics, to verify their eligibility. Due to the incidence of phishing from out of state and attempted repeat participants, the origins of IP addresses were also verified to ensure the validity of the participants. Participants were offered a $30 digital gift card for their time. The study was approved by the University of Illinois Chicago Institutional Review Board.

### Interviews

All interviews were conducted by Black and Latinx graduate students to promote participant comfort and candor.^34^ Interviews were conducted in a one-on-one, in-depth, semistructured format *via* telephone or Zoom software and were recorded and transcribed for analysis. No respondent requested an in-person interview. Verbal informed consent was obtained from each participant. Semistructured interviews lasted approximately 30–60 minutes.

### Data analysis

Interviews were transcribed by a professional transcription service and coded by authors (E.G. and K.B.) using Dedoose software (v9.6.004). The interviews were organized and analyzed thematically. Thematic analysis and codebook development began while data collection was ongoing. Thematic categories were developed based on the FRAME-IT constructs to guide analysis.^33^ The researchers used inductive and deductive coding to further analyze the transcripts.

An *a priori* sample of 15–25 participants was selected based on previous studies and the anticipated complexity of our research questions.^35–37^ Data were analyzed after completion of the 17th interview, and it was decided that inductive thematic saturation was reached.^38,39^

## Results

In total, 17 women were interviewed. The participants self-identified as Black or African American (*n* = 9), Hispanic/Latina/Latinx (*n* = 7), or “other” (*n* = 1). The participants’ ages ranged from 25 to 63, with a median age of 37. Participants reported various levels of completed education, ranging from “some high school” to “multiple graduate degrees.” Seven participants reported previous full completion of HPV vaccination; 64.7% (*n* = 11) self-reported up-to-date cervical cancer screening while only one participant had never been screened for cervical cancer (Table 1).

**Table 1.** **Community Member Characteristics and Demographic Information (***N* = 17)

Characteristic	Total *N* (%)
Age (median, range)	37 (25–63)
Race/ethnicity	
Black/African American	9 (52.9)
Hispanic/Latina/Latinx	7 (41.2)
Other	1 (5.9)
Education level	
Some high school	1 (5.9)
High school/GED (General Education Diploma)	2 (11.8)
Some college	4 (23.5)
Associate degree	2 (11.8)
College degree	7 (41.2)
Master’s degree	1 (5.9)
Relationship status	
Single	11 (64.7)
In relationship	3 (17.6)
Married	3 (17.6)
Employment status	
Full-time employee	8 (47.1)
Part-time employee	2 (11.8)
Self-employed	3 (17.6)
Unemployed	2 (11.8)
Other (disability)	2 (11.8)
Vaccinated for HPV (up-to-date^a^)	
Yes	7 (41.2)
No	10 (58.8)
Prior cervical cancer screening history	
Screened within 5 years	11 (64.7)
Not screened within 5 years	5 (29.4)
Never received screening	1 (5.9)

^a^ Up-to-date means three doses of the HPV vaccine if first administered after age 15 and two doses of HPV vaccine if administered before age 15.

Through analysis of the 17 interviews, patterns and themes emerged. Specifically, participants faced financial, logistical, and emotional barriers that prevented them from seeking regular, preventative health care. They also demonstrated support for a self-collected HPV testing method.

### Financial barriers to care

Financial constraints were identified as a substantial barrier to accessing cervical cancer screening in this study. Multiple participants mentioned the cost associated with obtaining cervical cancer screening, noting that the expenses included the cost of consultations with both primary care providers and gynecologists, and any additional tests or procedures that may be recommended during or after the screening process. For some participants, these expenses posed a financial burden, leading to trade-offs with other essential expenses, such as food or childcare, as discussed by the following participant:

*Well, it’s expensive. The consult is expensive…It would cost me to book an appointment with my primary care provider. And it would also cost me to book an appointment with my gynecologist. And then it would cost me for any [testing that is done] afterwards. And although I can pay for that, that would mean I don’t get to pay [for] other things that I need in order to survive. (Woman, Latinx, 27)*

This financial strain was particularly challenging for individuals who were students or parents with multiple responsibilities.

### Logistical barriers to care: Transportation, scheduling, and competing life priorities

Logistical barriers in transportation and scheduling were reported by many participants. It was cited as the main reason they did not seek regular preventative care in general. Regarding follow-up care, one participant said, “If you have transportation, it’s okay [to take the time for follow-up care]” (Woman, Black, 30).

In addition, many participants discussed scheduling issues as a major reason they did not seek regular preventative care. One participant discussed a lack of availability in appointments that she could attend, explaining that for many of the appointments, “most of the time [she] was just working” (Woman, Black, 59).

Scheduling and attending regular health care, such as cervical screening, was particularly difficult for our participants living on the south side of Chicago. According to one participant, health care clinics on the south side have closed continuously without being replaced by new ones. For this participant, the self-collected HPV testing would ease some of the burden of identifying local clinic locations that offer other preventative care. She explains:

Yes. Because us Black people, they get so…left behind. Then, they have to suffer in private. So, I think [self-collected HPV DNA testing] is very important. (Woman, Black, 30)

Participants also cited prioritization of other aspects of life as a barrier to cervical cancer screening. This included work commitments, family responsibilities, and various life events. Participants acknowledged that, at certain points in their lives, factors such as work and family took priority over health screenings. One participant described her difficulties in seeking regular preventative care as follows:

*And at that point, I think it was just other priorities in life, right? Like, work, family—there was just so much more happening, so I don’t even think that it was—from the ones that you listed, I don’t really think those were registering in my brain. It was just like other things were happening, and so those to me were a priority. (Woman, Latinx, 36)*

Issues with scheduling and transportation reported by the participants underscored the need for screening methods that are convenient and fit into individuals’ lives to ensure they can access screening and receive timely treatment when necessary.

### Logistical barriers to care: Establishing care with a physician

Participants reported difficulty establishing care with health care providers. Particularly for participants using state health care (Medicaid), finding available clinics accepting new patients in their desired specialty was difficult. One participant described why she missed her last screening:

*I think I was still trying to establish care with an OB/GYN. I have an Internal Med. physician and I kinda wanted to separate the two. (Woman, Latinx, 25)*

As this participant indicated, choosing practitioners often leads to barriers such as long wait times for scheduling and establishing care. Another participant explained that the low availability of appointments is prohibitive to regular care:

*Now, also the appointment sometimes…if they refer you to an OB/GYN, the appointments are like you have to wait three months before you can actually get an appointment. (Woman, Other, 37)*

Alternative screening methods that do not require an in-person appointment with a physician could address these barriers.

### Emotional barriers to care: Experiences of trauma and anxiety

For some participants, past experiences of trauma and anxiety were substantial barriers to seeking cervical cancer screening. One participant revealed a history of sexual assault and molestation, which had a profound impact on her perception of medical procedures, including cervical cancer screening. The fear of the unknown and the potential for discomfort or distress during the examination were cited as reasons for avoidance. Participants also cited their past experiences with cervical cancer screening and pelvic exams as a major factor in their hesitancy to seek regular preventative care. Several participants discussed experiences of physical discomfort and pain during these exams. One participant stated:

*But I also think it’s uncomfortable. Like a lot of people procrastinate to go in to get a testing only because of how uncomfortable and exposing [a pelvic exam is]. (Woman, Other, 37)*

Another participant commented on the discomfort from the perspective of being lesbian:

*I’m a lesbian so the feel of any type of anything going there in that area it’s going to be uncomfortable; it’s going to be something that nobody wants to experience, especially if it’s not something that you’re used to doing or having done. (Woman, Black, 25)*

These experiences have varied origins but the question of discomfort has important implications for how cervical cancer screening could be improved.

Despite some participants’ reluctance to go into a clinic and have an in-person exam, all of the participants confirmed that they would seek follow-up care should their self-screening HPV test results yield inconclusive, “troubling,” or positive results. One participant explained that:

*Like I said, it was uncomfortable. That’s probably why I don’t do it often. But if I have to do a follow-up and it’s a positive test or whatever, I just do what I gotta do. You know what I mean? Because I need to follow up ... for whatever reason to make sure everything is okay. (Woman, Black, 39)*

According to participants, HPV self-collection would remove the initial hurdle of seeking out health care for a test, which many found uncomfortable. This would allow participants to focus on the potential outcome and next steps when testing results are received. In addition, the self-collected HPV testing option allowed these participants to perform the test at a time and location that was most comfortable and agreeable to them and their schedules.

### Preferred self-collected HPV testing methods

The participants overwhelmingly supported the self-collected HPV testing method as an alternative to traditional cervical cancer screening and thought it would address the current barriers they face. For participants whose primary barrier was pain and discomfort, self-collected HPV testing provides a patient-centered alternative: “For privacy reasons, comfortability reasons, those would be the main reasons why [I would try the self-swab method]” (Woman, Black, 25). Many also expressed the need for and interest in a cervical cancer screening method that is more affordable and accessible:

*I would 100% get myself taken care of in that way because women die from this. And I don’t want to be that woman. I don’t want to be part of that percentage that dies because they didn’t take care of themselves. And it’s not their fault that they died…or that they have cervical cancer…But either the tools to get taken care of, getting checked up early were not there, or they couldn’t afford it like I can’t. (Woman, Latinx, 27)*

Another expressed the need for timely screening and saw self-collected HPV testing as a means to get more women up to date:

*My body has changed so much since I’ve gotten older, and I find it a little more difficult, whereas I used to work out all the time. I find that it would be more difficult for me to be consistent [staying up to date with screening]. So, I would want to know. I wouldn’t want to be around here at the last minute, you know? I want to know if there is something going on in my body. (Woman, Black, 63)*

Other participants remarked on the convenience of being able to complete HPV testing at home, as opposed to a clinic, which presents barriers to scheduling:

*It would be really convenient for me to just get it and send [from] home. And I don’t know how long it’ll be before I set up my own appointment to get it done. And so, if it was available today, I would say, “Mail it out today before 5:00.” (Woman, Latinx, 36)*

Most participants would prefer to complete self-collected HPV testing at home, followed by an in-clinic location, or a community center. One participant explained why she would prefer testing at home:

*If you can do it from home, and also not being able to have appointments right away, and if you have this provided to you and you can do it at home without having to see a provider, I think that’s a great idea. (Woman, Latinx, 37)*

Participants expressed interest in instructional pamphlets, visual aids, community health worker presence or support, and provider presence or support while performing self-collected HPV testing. Participants were interested in these resources to ensure testing accuracy, indicating they would be helpful “to get some guidance [to] do it the right way” (Woman, Latinx, 46). Overall, participants were supportive of the self-collected HPV testing method because it addresses the barriers to screening they face and their desire to ensure they can conduct the testing with accuracy.

## Discussion

The results of this study introduce the critical role that self-collected HPV testing could play in addressing disparities in cervical cancer screening, particularly among Black and Latinx women in Chicago. The findings indicate a strong preference for self-collected HPV testing among these individuals, with the potential to overcome several barriers to care, including costs of appointments, transportation, scheduling, provider access, and lived experiences of anxiety and trauma. Many of these barriers align with the social determinants of health^40^ that must be addressed through social support and innovation, rather than medical intervention. Efforts to improve the ease of scheduling, increase provider availability, improve access to transportation, and improve the quality of care are necessary to begin to address these social determinants of health. This final point especially highlights the importance of providing patient-centered and trauma-informed care, as well as less-invasive screening options like self-collected HPV testing, to make screening more accessible and comfortable for individuals with a history of trauma and anxiety.

The findings are consistent with other U.S.-based studies that explored the efficacy of HPV self-collection in underscreened communities. Primarily Hispanic participants of a 2008 study assessing the sensitivity and specificity of self-collected HPV testing compared with physician-collected samples in Santa Ana, CA, found the at-home self-collection kit to be easy to use and more convenient than going to the doctor’s office.^41^ These sentiments were echoed in studies completed in rural North Carolina, Appalachian Ohio, and the Mississippi river delta, where most participants were African American or Black.^24,26,31^ All of the 79 participants in Appalachian Ohio preferred self-collection than going to the doctor’s office.^26^ Moreover, uptake was high in a trial of community health worker-delivered or mailed self-collected HPV testing kits in South Florida among the Hispanic and Haitian immigrant communities, where roughly three quarters of participants completed self-collection in both groups.^42^ Only 9% of participants in the Appalachian Ohio study and 16% of participants in the North Carolina study, when specifically prompted to discuss their difficulties with the self-collection test, were concerned about performing the self-collection incorrectly.^24,26^ This was also mentioned by participants of this present study; however, there is no further information in the prior studies or commonalities with the present to conclude anything about the types or groups of participants who shared this concern.

### Limitations

The sample size for this study (*n* = 17) was relatively small and was recruited from the entire Chicago metropolitan area and may not represent the experiences of populations living exclusively in the south and west sides of Chicago. However, sampling methods and participant recruitment were designed to reach Chicago population’s most affected by cervical cancer incidence to better inform future interventions. These findings may not be applicable to marginalized populations in different metropolitan or rural areas. In addition, conducting interviews virtually may introduce information bias due to potential technical issues or lack of nonverbal cues and also potentially increase social desirability stemming from the respondent’s remote setting.

The self-collected HPV testing method was seen as a potential answer to specific barriers by facilitating improved access to cervical cancer screening. However, while this testing method addresses certain obstacles, it does not solve the overarching issue of barriers to health care that could impact follow-up care, underlining the need for continued efforts to enhance overall health care accessibility. Future implementation of self-swab screening may also face logistical challenges; the distribution and return of test kits, result communication, follow-up scheduling, and the necessity of a billable encounter with a health care provider for follow-up are all potential barriers to care. However, these barriers have been successfully addressed in New Zealand *via* the distribution of tests *via* community health centers, mobile outreach services, and an accessible database of providers who offer the tests and would be reasonable to implement here.^43^ While self-collection may not be the sole answer to barriers in cervical cancer screening, it may serve as one tool in a kit of several possible solutions to reach different populations with different needs and insurance coverage. Alternatively, one may argue that more effort could be made to address existing structural barriers to care on their own.

Notwithstanding these limitations, our study elucidated the particular barriers experienced by Chicago women when seeking cervical cancer screening, which may be applicable to other parts of the country. Our study also highlighted the importance of recognizing past pain and trauma as a barrier to screening, which was not extensively explored by other studies.

### Practice and policy implications

These findings strongly demonstrate the support for self-collected HPV testing among women because of its ability to address the aforementioned barriers and provide a more convenient option for cervical cancer screening. This is especially relevant with the recent FDA approval of self-collected HPV testing, which may also soon be integrated into screening guidelines and recommendations by major organizations such as the U.S. Preventive Services Task Force.

Changes to cervical cancer screening programs at the organizational level that accommodate this self-collected HPV testing method may lead to more regular screening, especially among underscreened populations. This may be relevant not only for Black and Latinx women but also for other marginalized populations including lesbian, gay, and bisexual cisgender women, where screening has been shown to be lower in Chicago in particular.^44^ In general, self-collected HPV testing programs should take a trauma-informed approach and utilize community-focused techniques such as involving community health workers or mailed kits to improve up-to-date screening prevalence. Health care organizations should pay particular attention to the needs and history of the communities they serve when designing policies and procedures to implement HPV self-collection within their existing cervical cancer screening programs.

To address financial barriers, self-collected HPV testing should also be covered by health insurance. The Federal Department of Health and Human Services should include the self-collection method in the preventive care benefits for women required by all Health Insurance Marketplace plans.^45^ The Centers for Medicare & Medicaid Services should also cover self-collected HPV testing through Medicare and Medicaid, without restrictions on co-testing with a Pap test (cytology), since patients are unable to perform Pap tests on their own.^46,47^

## Conclusions

Addressing the disparities in cervical cancer screening, incidence, and mortality among Black and Latinx women in Chicago is imperative. This study demonstrates that self-collected HPV testing offers one promising solution that could combat barriers to care.

Moving forward, the implementation of self-collected HPV testing in clinical practice, alongside person-centered, trauma-informed, and targeted community outreach and education, has the potential to significantly increase rates of cervical cancer screening and decrease cervical cancer incidence and mortality among marginalized populations. Efforts should be made to ensure that this innovative approach becomes a routine part of cervical cancer screening programs, and that it is covered by insurance, ultimately, preventing cases of cervical cancer and reducing health care disparities in Chicago and beyond.

## Abbreviations Used

BCBSIL: Blue Cross Blue Shield of Illinois
FDA: Food and Drug Administration
GED: General Education Diploma
HEPP: Health Equity Pilot Program
HPV: human papillomavirus
NL: non-Latinx
