# Supplementary material for: The Acceptability and Feasibility of Self-Collected HPV Testing for Cervical Cancer Screening Among Black and Latinx Women in Chicago: Perspectives from the Community
Source: Womens Health Rep (New Rochelle). 2024 Sep 30;5(1):735–43. doi: 10.1089/whr.2024.0102 (PMC11512086; doi:10.1089/whr.2024.0102)
Supplement: Supplementary Data S1 [file whr.2024.0102_supp_datas1.pdf]

The acceptability and feasibility of self-collected hrHPV tests for cervical cancer screening in Black and Latinx women in Chicago: perspectives from the community and their healthcare providers

Key Informant Interview Guide: Individuals with Lived Experience

Research questions: What do women and people with cervixes think about self-swabbing for HPV?

Interview guide:

My name is **[NAME]** and I'm calling from the UIC Center for Research on Women and Gender for our scheduled interview regarding research on self-swabbing for cervical cancer. -- Is this still a good time to talk?

Great! You have been asked to participate because you identify as Black or Latinx, are between the ages of 25 and 65 years old, can communicate in English, and have never had a hysterectomy.

Our previous communication included a Consent and Information sheet that detailed the parts of the study. Do you have any questions about this sheet, the study or your participation before we get started?

Your participation in this interview is voluntary and all your responses are confidential and will be reported collectively. You can skip any question and can stop the interview at any time. The interview should last about 45 minutes. After completing the interview, we will send you a $30 digital Target gift card via email for your participation.

Do you agree to take part in the interview? Do you have any questions before we start? Do I have your permission to record the interview?

Before we start, I want to ask about some background information about your history and experience with cervical cancer screening.

Part 1: History of Cervical Cancer Screening and Identifying Barriers

1. Have you ever been screened for cervical cancer before? This is often called a pap smear.
2. If applicable: What was your experience with your first pap smear like?
    a. Probe into: discomfort/traumatic/pain
3. Have you ever had an abnormal/positive cervical cancer screening test?
4. What are your thoughts on cervical cancer screening? Why/why not did you get screened?

5. Do you think getting screened is necessary for your health?
    a. If no: use US Preventative Services Taskforce information page to provide brief education
6. If applicable: When was the last time (in years) that you were screened for cervical cancer?
7. What prevented you or made it difficult for you to get screened for cervical cancer?
    a. Prompts: your earlier experience, transportation, scheduling difficulties, affordability of the test

Thank you for sharing your experiences with me.

Part 2: Description of Self-Swab Method

Now I am going to describe a new method for cervical cancer screening, and I want to hear your thoughts about it.

Description: For this screening method, you will use a swab (similar to a long q-tip), insert it into your vagina, swirl it around, and then put the swab in a small tube (show picture). This swabbing method is similar to the self-swab for STIs or a COVID test (nose) that you may have done in the past.

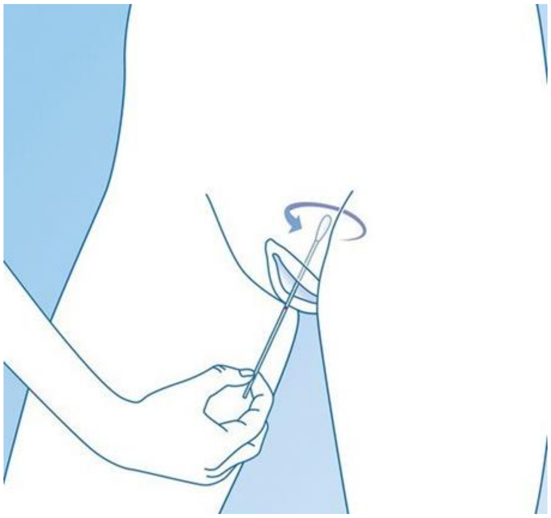

The self-swabbing method is highly effective and can discover more pre-cancerous changes due to HPV (human papilloma virus) then a pap smear. Compared to a pap smear that includes laying on a table and an exam with a speculum, the self-swab screening method is something that you can do on your own or choose to do the swab with assistance from a medical professional or community health worker.

8. Do you have any questions about what I just described?

Part 3: Probes to Address Barriers Mentioned in Part 1

9. Barrier: Invasiveness/Abuse
    a. You mentioned that your (prior experience with abuse, uncomfortable with invasiveness of test with provider) is a barrier.
        i. The self-swab screen is a test that you can do yourself at home or, if desired, you have the choice to have a provider or community health worker assist you. Would doing the self-swab yourself make you feel more comfortable with getting tested?
10. Barrier: Transportation/Difficulty scheduling appointment/Lack of Time
    a. You mentioned that (finding transportation to/from the clinic; appointment time that works with your schedule/finding childcare) is a barrier.
        i. Would receiving/sending your kit back by mail or completing the self-swab at a local community center increase the likelihood of getting tested?
11. Barrier: Painful/Discomfort
    a. You mentioned that getting a speculum exam was very painful and made you not continue to get tested. The self-swab is much gentler and shouldn't be painful – it just includes swirling a long q-tip in your vagina. Does this sound like something that would interest you?
12. Barrier: Cost/Affordability of test
    a. You mentioned that you had issues paying for the HPV testing. As the self-swab is a preventative health screening measure, it would be covered by insurance (even Medicaid, Medicare, required by Affordable Care Act to cover). With insurance coverage for a self-swab, would that increase the likelihood of getting tested?
13. Barrier: Concerns about accuracy of test method
    a. You mentioned that the accuracy of the test is a concern for you. The self-swab testing method is as accurate as the current testing method (pap smear with speculum). Does knowing this make you feel more comfortable with using the self-swab method?

If Not Covered in Interview, Ask the Following Questions:

14. The self-swab method could be done at a location of your choosing. Do you have a preference on where to complete the self-swab: home, clinic, community center?
15. Would you want support in completing the self-swab?
    a. Prompt: a provider or community health worker available to give instruction
16. What supports would you need to feel comfortable completing the self-swab?
    a. Prompt: pamphlet, visual instruction, access to a provider or community health worker to ask questions

Part 4: Test Results

17. How would you prefer to receive your test results?
    a. Prompt: phone call, text, clinic portal, email, in person
18. Does the method you'd prefer for receiving your test results differ based on whether you have a positive or negative result?
19. If you received a positive test result, how likely would you follow-up with your provider about next steps?
    a. Prompt: work with provider for more testing
20. The HPV self-swab test is highly accurate. If you get a positive result, you will need to do a second test in-person at the clinic. This second test might also be positive and require follow-up or it may come back as negative. How does potentially needing more in-person testing make you feel?
21. Based on the information that I have shared with you today, if self-swabbing for HPV were offered to you, would you get screened?
    a. Why/why not?

Part 5: Demographics

Thank you for sharing your experiences with me. Before we end this interview, I would like to ask a couple of demographic questions. <u>Just as a said at the beginning of this interview, you can skip any of these questions. These demographic questions will not be tied to your responses.</u>

22. Have you ever been vaccinated for HPV before?
23. What race or ethnicity do you identify with?
24. Did you grow up outside of the United States?
25. In what city do you currently live?
    i. Note: if Chicago, please name the neighborhood.
26. Which gender do you identify with?
27. Do you identify as part of the LGBTQ+ community?
28. What is your current relationship status?
29. What is your highest level of education?
30. What is your current employment status?
31. How old are you?

Thank you for taking part in this phone interview. Your contributions <u>will help us understand the best way to implement self-swabbing for cervical cancer screening.</u>

32. Do you have any questions for me before we end?
